# Epidemiology and economic burden of selected rare genetic diseases in Germany – a claims database study

**DOI:** 10.1186/s13023-025-04147-8

**Published:** 2025-11-28

**Authors:** Marion Ludwig, Marco Alibone, Raeleesha Norris, Josephine Jacob, Vukasin Viskovic, Anja Cengia, Dominik Obermüller

**Affiliations:** InGef - Institute for Applied Health Research Berlin GmbH, Otto-Ostrowski-Str. 5, 10249 Berlin, Germany

**Keywords:** Rare diseases, Administrative claims, Healthcare, Epidemiology, Health care costs

## Abstract

**Background:**

Research on rare genetic diseases is challenging due to their low prevalence and complex clinical manifestations. Despite their individual rarity, the cumulative burden of these diseases is substantial. Larger claims databases could enable a quantification and characterization of patients with rare diseases and contribute to determining their specific healthcare needs. This study examines the prevalence, comorbidities, and annual direct costs of Huntington’s disease (HD), Beta-Thalassemia (BT), and Spinal Muscular Atrophy type 1 (SMA type 1) in Germany.

**Methods:**

Utilizing anonymized claims data from the InGef research database, we conducted a cross-sectional analysis for each disease from 2017 to 2023. Patients and comorbidities were identified based on ICD-10-GM-codes. Prevalence was extrapolated to the German population. Costs were analyzed as direct costs per patient and by sector incurred.

**Results:**

Prevalences for the selected diseases were 6.65 (HD), 12.81 (BT), and 1.24 (SMA type 1) patients per 100,000 persons in Germany in 2023. The most frequent comorbidities identified are strongly related to the specific disease. For instance, in 2023, among the most common diagnoses were depression in HD patients (38.48%), dorsalgia in BT patients (37.03%), and acute upper respiratory infections in SMA type 1 patients (45.19%). Average total direct costs in 2023 amounted to 9,527.89 € for HD, 6,656.27 € for BT and 144,585.02 € for SMA type 1, respectively.

**Conclusion:**

Claims data enable robust identification and characterization of rare disease populations. The findings on prevalences and comorbidities align with existing literature and support the use of such data for treatment planning and post-marketing studies.

**Supplementary Information:**

The online version contains supplementary material available at 10.1186/s13023-025-04147-8.

## Background

 Rare diseases, although individually uncommon, collectively affect a substantial number of individuals and are associated with considerable morbidity, reduced quality of life, and high socioeconomic burden for both families and healthcare systems. In many countries, including Germany, comprehensive and up-to-date epidemiological data on rare diseases are limited. Existing evidence is often fragmented, based on small cohorts, or derived from disease-specific registries with variable completeness and coverage [1, 2]. 

Routinely collected data from the statutory health insurance (SHI) system represent a largely untapped resource for rare disease research. These data include detailed information on diagnoses, comorbidities, pharmacotherapy, and healthcare utilization, enabling robust assessments of disease burden and care needs. Moreover, SHI data can support post-marketing surveillance and inform the development of evidence-based clinical guidelines [3]. 

Recent regulatory developments have strengthened the potential of SHI data for rare disease research. Since 2023, the standardized coding of rare diseases in inpatient care using the identification number Alpha-ID-SE, which incorporates ORPHAcodes, has been mandated in Germany. Although the implementation was not yet extended to outpatient care and other sectors it might contribute to the diagnostic precision of SHI data and facilitates more consistent identification of rare disease cases in the future [4]. 

This study explores the potential of SHI data for the epidemiological assessment and health services research of rare diseases in Germany, highlighting both opportunities and remaining limitations in current data infrastructures analyzing rare diseases. Three genetic conditions with specific ICD-10-GM codes serve as use cases to provide recent data on prevalence, comorbidities, and associated healthcare costs.

## Methods

### Data source

The InGef research database comprises anonymized claims data on the utilization of healthcare services of approximately 10 million insured individuals from 50 German statutory health insurance funds (SHIs) currently contributing data [5]. The dataset includes insured persons from all federal states. All patient- and provider-level data are anonymized in accordance with German data protection regulations and federal legislation. Therefore, approval from an ethics committee was not required. Currently, the data are longitudinally linked over a period of ten years. In addition to sociodemographic information, the database contains data on outpatient services and diagnoses; hospital data including admission dates, main and secondary diagnoses, as well as performed surgeries and procedures; [6] information on prescribed medications; [7] and data on medical aids, remedies, and sick leave data. Clinical diagnoses are coded according to ICD-10-GM (German modification of the 10th revision of the International Classification of Diseases).

### Study design and study population

This retrospective observational study was conducted using data from calendar years 2017 to 2023. The insured population was analyzed cross-sectionally for each individual study year. Individuals were included if they were fully observable either during the respective study year or from birth until December 31 or death of the same year. All analyses were conducted separately for the rare diseases under investigation: Huntington’s Disease (HD), Beta-Thalassemia (BT), and Spinal Muscular Atrophy type 1 (SMA type 1).

The prevalent rare diseases investigated were defined by the documentation of the corresponding ICD-10-GM diagnosis code (HD: G10; BT: D56.1; SMA type 1: G12.0). Diagnoses were considered if recorded either as a main or secondary diagnosis in the inpatient setting or as a confirmed diagnosis in the outpatient setting. To improve diagnostic certainty and reduce the risk of misclassification based on single, potentially erroneous claims, we applied the M2Q (minimum two quarters) criterion. According to the M2Q definition, an outpatient diagnosis was only considered valid (i.e., indicating a prevalent case) if it was: (a) followed by a confirmed outpatient or inpatient diagnosis in a different calendar quarter within the same year, or (b) documented by a different physician within the same calendar quarter. This approach ensures that the diagnosis is supported by repeated and/or independent documentation across time or providers, enhancing the specificity of case identification based on routine data. Prevalence was calculated for each study year by dividing the number of existing cases by the total study population and is presented per 100,000 persons along with 95% confidence intervals. Furthermore, prevalence estimates were extrapolated to the German population using population estimates provided by the Federal Statistical Office of Germany (DESTATIS) [8].

The most common comorbidities among included patients were identified based on at least one confirmed outpatient diagnosis or inpatient main or secondary diagnosis (ICD-10-GM codes at the three-digit level) within the respective calendar year. Costs incurred by the SHI were analyzed as direct costs per patient, both in total and stratified by healthcare sector (i.e. outpatient, inpatient, medication, and aids and remedies), and were presented using descriptive statistics. Cost data were further log₁₀-transformed to improve comparability and visualized using boxplots stratified by disease, year, and sector. For data protection reasons, no measures are reported for strata with less than five patients.

### Trend analysis

To evaluate temporal trends in disease prevalence and mean annual total costs, Joinpoint regression analysis was performed using the Joinpoint Regression Program (Version 5.4.0; National Cancer Institute, USA). The analysis covered the study period from 2017 to 2023. The Annual Average Percentage Change (AAPC) and corresponding 95% confidence intervals (CIs) were calculated for each outcome. AAPC values were considered statistically significant if the 95% CI did not include zero (*p* < 0.05). The final model selection was based on the Monte Carlo permutation method. For prevalence trends, the analysis was based on the annual prevalence estimates derived from the InGef research database and extrapolated to the German population. For cost trends, the analysis was based on mean annual total costs per year.

## Results

### Prevalence

**Fig. 1:**
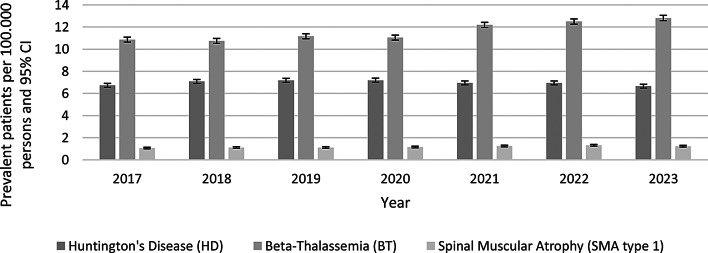
Prevalence of selected rare diseases in Germany from 2017 to 2023. The bar chart displays the annual prevalence (per 100,000 persons) of Huntington’s disease (HD), Beta-Thalassemia (BT), and Spinal Muscular Atrophy type 1 (SMA type 1), including 95% confidence intervals for the German population

Out of the 7,297,840 continuously insured persons in 2023, 564 patients with Huntington’s Disease (HD), 1,072 patients with Beta-Thalassemia (BT), and 104 patients with Spinal Muscular Atrophy type 1 (SMA type 1) were identified based on the respective ICD-10-GM diagnosis code. This corresponds to prevalences of 7.73 (HD), 14.69 (BT), and 1.43 (SMA type 1) patients per 100,000 persons in 2023 within the InGef research database. Prevalences directly standardized to the German population for the selected diseases over the analyzed data years are shown in Fig. 1; Table 1.

To assess trends in disease prevalence over time, Joinpoint regression analyses were conducted for HD, BT, and SMA type 1 using the standardized prevalences for the years 2017 to 2023. A statistically significant increasing trend was observed for both SMA type 1 (AAPC: 3.243%, 95% CI: 1.54–5.11, *p* < 0.000001) and BT (AAPC: 3.241%, 95% CI: 2.03–4.58, *p* < 0.000001). For HD, Joinpoint regression identified one significant change in trend in 2019 (Joinpoint). From 2017 to 2019, the standardized prevalence increased significantly with an Annual Percentage Change (APC) of + 3.67% (95% CI: 0.76–6.86; *p* = 0.004). This was followed by a significant decline from 2019 to 2023 (APC: − 1.94%, 95% CI: − 4.32 to − 1.13; *p* = 0.001). Over the full study period, the AAPC was − 0.10% (95% CI: − 1.03 to 0.76; *p* = 0.652), indicating no overall significant trend.

Table 2 displays the demographic characteristics of the respective rare diseases for the most recent year 2023 based on the InGef research database. Median age was 57 years (Q1-Q3: 50–65) for HD, 43 years (Q1-Q3: 29–57) for BT and 19 years (Q1-Q3: 4–54) for SMA type 1. The patient distribution by age group and the proportion of females remained constant over the observed data years for the reported diseases.

**Table 1:** Displays the annual prevalence (per 100,000 persons) of huntington’s disease (HD), Beta-Thalassemia (BT), and spinal muscular atrophy type 1 (SMA type 1), including 95% confidence intervals for the German population

	Prevalent patients per 100.000 persons (95% CI)						
Disease	2017	2018	2019	2020	2021	2022	2023
HD	6,73 (6,55–6,91)	7,08 (6,90–7,26)	7,18 (7,00–7,37)	7,19 (7,01–7,38)	6,95 (6,77–7,13)	6,95 (6,77–7,13)	6,65 (6,48–6,83)
BT	10,86 (10,64–11,09)	10,75 (10,53–10,97)	11,16 (10,93–11,39)	11,04 (10,82–11,27)	12,19 (11,95–12,43)	12,49 (12,26–12,73)	12,81 (12,57–13,05)
SMA type 1	1,07 (1,00–1,14)	1,11 (1,04–1,19)	1,12 (1,05–1,19)	1,16 (1,09–1,23)	1,25 (1,17–1,33)	1,32 (1,24–1,40)	1,24 (1,16–1,31)

**Table 2:** Demographic characteristics of patients with huntington’s disease (HD), b-Thalassemia (BT) and spinal muscular atrophy type 1 (SMA) for the year 2023

	HD		BT		SMA	
	*N*	%	*N*	%	*N*	%
**Total patients**	564	100	1072	100	104	100
0–20 years	< 5	-	181	16.88	55	52.88
21–50 years	148	26.24	506	47.20	19	18.27
>50 years	412	73.05	385	35.91	30	28.85
**Male**	271	48.05	458	42.72	55	52.88
**Female**	293	51.95	614	57.28	49	47.12

As an excerpt for the entire analysis, results for the current year 2023 are presented. The most frequently documented comorbidities were largely specific for each disease (Table 3). Depressive episodes (38%) and abnormalities of gait and mobility (31%) were most frequently reported among patients with HD, while acute upper respiratory infections of multiple and unspecified sites were most prevalent in patients with BT (38%) and SMA type 1 (45%), followed by dorsalgia (BT, 37%) and dependence on enabling machines and devices (SMA type 1, 38%).

**Table 3:** Most frequent comorbidities based on ICD-10-GM (in- and outpatient setting) in 2023. Abbr. Huntington’s disease (HD), Beta-Thalassemia (BT) and spinal muscular atrophy type 1 (SMA type 1)

HD (*n* = 564)	BT (*n* = 1072)	SMA type 1 (n=104)
Depressive episode *n* = 217 (38.48%)	Acute upper respiratory infections of multiple and unspecified sites *n* = 408 (38.06%)	Acute upper respiratory infections of multiple and unspecified sites *n* = 47 (45.19%)
Abnormalities of gait and mobility *n* = 178 (31.56%)	Dorsalgia *n* = 387 (37.03%)	Dependence on enabling machines and devices *n* = 40 (38.46%)
Essential (primary) hypertension *n* = 169 (29.96%)	Essential (primary) hypertension *n* = 318 (29.66%)	Scoliosis *n* = 39 (37.50%)
Other mental disorders due to known physiological condition *n* = 166 (29.43%)	Disorders of refraction and accommodation *n* = 270 (25.19%)	Respiratory failure, not elsewhere classified *n* = 37 (35.58%)
Aphagia and dysphagia *n* = 145 (25.71%)	Abdominal and pelvic pain *n* = 247 (23.04%)	Other disorders of muscle *n* = 26 (25.00%)

### Direct costs

**Fig. 2:**
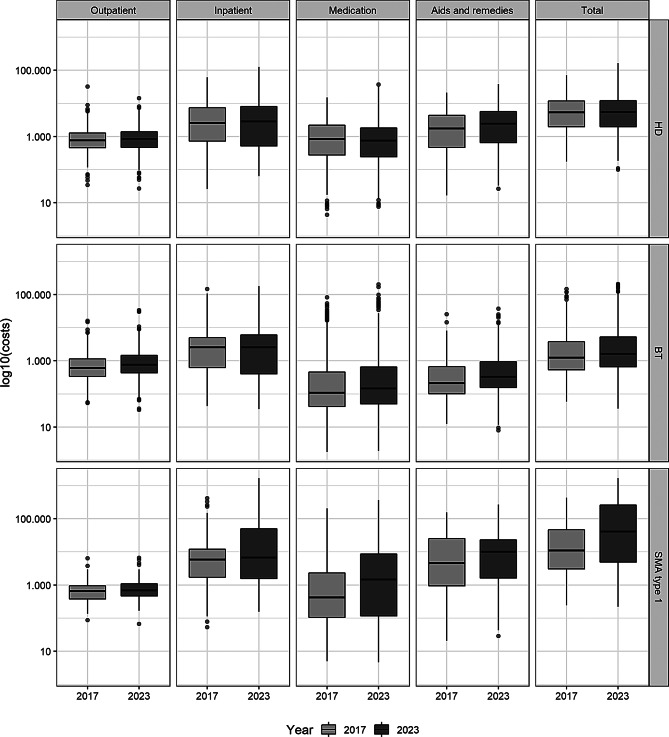
Boxplots of log₁₀-transformed direct costs (in €) per patient for the three diseases in 2017 and 2023, shown across the cost sectors (Outpatient, Inpatient, Medication, Aids and Remedies) and total. Each box represents the median and interquartile range (IQR); whiskers extend to 1.5× IQR, and outliers are shown as individual points

In 2017, the mean total direct medical costs per patient amounted to €46,286.70 for SMA type 1, €4,605.13 for BT and €8,375.62 for HD. By 2023, these had risen to €144,585.02, €6,656.27, and €9,527.89, respectively. The most pronounced increase was found for SMA type 1 (3.6-fold increase from 2017 to 2023), which is mostly due to a strong increase in inpatient and medication costs. Figure 2 presents the distribution of mean annual costs per patient by sector (outpatient, inpatient, medication, aids and remedies, and total) for each disease and year, presented as boxplots. Cost distributions were highly variable for all diseases, indicating substantial heterogeneity among patients. To improve readability and comparability across cost sectors and disease groups, costs are shown on a logarithmic (log₁₀) scale. Descriptive statistics of costs incurred by sector for the years 2017–2023 are given in additional file 1.

Between 2017 and 2023, mean annual total costs per patient showed varying trends across the three diseases. For SMA type 1, costs increased significantly, with an AAPC of 22.3% (95% CI: 2.2% to 46.3%; *p* = 0.029). Similarly, BT exhibited a significant upward trend with an AAPC of 6.0% (95% CI: 4.0% to 8.1%; *p* < 0.000001). In contrast, the increase observed in HD, with an AAPC of 2.8% (95% CI: − 0.05% to 5.9%; *p* = 0.054), was not statistically significant. No joinpoints were detected in the analyses for any of the rare diseases, indicating that the trends over the observation period were linear and did not change direction.

## Discussion

This study provides a comprehensive overview of the prevalence, comorbidities, and healthcare costs associated with three rare diseases—Huntington’s disease (HD), Beta-Thalassemia (BT), and Spinal Muscular Atrophy type 1 (SMA type 1)—using claims data from the InGef research database. The findings offer important insights into the epidemiology and economic burden of these conditions and underscore the need for improved surveillance and data availability in the field of rare diseases.

Prevalence estimates observed in this study are largely consistent with previously published data where available. Minor deviations from existing literature may be explained by differences in study design, data sources, or temporal factors, as disease prevalence can shift over time. Moreover, in contrast to ORPHA codes, the ICD-10-GM classification lacks the necessary granularity and does not provide the level of detail required to distinguish between specific rare diseases or even subtypes of disorders. For HD, a recent meta-analysis reported a pooled prevalence of 4.8 per 100,000, with higher rates in Europe and North America [1]. In Germany, a previous estimate indicated a prevalence of 9.3 per 100,000 during 2015–2016 [9]. Our study reports a prevalence of 6.65 per 100,000 in 2023, aligning well with this range and supporting the validity of the findings.

Despite advances in treatment and the implementation of newborn screening for SMA type 1 in Germany, data on the epidemiology and disease burden of SMA type 1 remain limited. A review by Verhaart et al. estimated a prevalence of 0.28 per 100,000 for SMA type 1, noting substantial regional variation, the frequent reliance on outdated data sources and studies conducted before the approval of effective therapies [2]. These early estimates are likely to reflect lower survival rates and reduced diagnostic awareness. In contrast, the prevalence of 1.24 per 100,000 observed in our study may thus, also reflect increased survival due to novel therapies and earlier diagnosis through screening programs [10]. 

BT remains one of the most common monogenic disorders globally, with particularly high prevalence in the Mediterranean, Middle East, and parts of Asia. In Germany and other parts of Central Europe, the number of cases has risen over recent decades, likely driven by migration [11–13]. However, robust data on BT in Germany are scarce. Our study presents the first claims-based prevalence estimates for BT (all subtypes) in Germany, showing an increase from 10.86 per 100,000 in 2017 to 12.81 per 100,000 in 2023. This trend highlights the growing healthcare relevance of BT and the need for continued monitoring and tailored healthcare planning.

The Joinpoint analysis revealed significant annual increases in standardized prevalence for SMA type 1 and BT of about 3.24% each from 2017 to 2023, likely reflecting improved diagnosis, treatment, and survival—especially with recent novel therapies for SMA type 1. For HD, overall prevalence remained stable, but a significant increase from 2017 to 2019 was followed by a decline through 2023. These fluctuations may result from changes in coding or diagnostic practices, possibly influenced by the COVID-19 pandemic’s impact on healthcare utilization, though random variation cannot be excluded.

Demographic characteristics observed for HD, BT, and SMA type 1 were consistent with existing literature. Median ages for HD (57 years) [14, 15], BT (43 years), and SMA type 1 (19 years) are consistent with the age profiles described in previous studies [16–18]. In contrast to BT and SMA type 1, HD typically presents in adulthood, with nearly all prevalent cases observed in individuals aged 21 years or older.

Across all three diseases, frequently observed comorbidities were closely related to the underlying condition, confirming the high disease burden experienced by affected individuals. Beyond the core symptoms of the index disease, patients commonly present with a wide range of additional health impairments that require independent clinical management or necessitate modifications to therapeutic strategies, especially in multimorbid patients.

Despite the limited lifespan of SMA type 1, patients face substantial clinical burden, with early onset of bulbar, respiratory, and nutritional complications requiring intensive medical care [19, 20]. Even under treatment, comorbidities and care needs remain significant. Real-world data from patients receiving disease-modifying treatments show that many patients continue to require non-invasive ventilation, feeding support, and experience frequent hospitalizations—averaging over nine admissions per patient-year in one Australian study [21]. Gene replacement therapy has improved clinical outcomes, especially when administered early. Registry data, such as from the RESTORE cohort, report motor function gains and potential for prolonged survival [22]. However, long-term support needs persist. From a health system and family perspective, SMA type 1 imposes a high caregiving burden. In a European caregiver study, the average informal care time reached 10 h per day—6.9 of which were typically provided by the main caregiver [23]. These findings emphasize that, despite advances in therapy, substantial resource utilization and caregiver strain persist, reinforcing the need for integrated support systems.

For Huntington’s disease (HD), progressive neurodegeneration leads to both motor and non-motor symptoms, including psychiatric and cognitive impairment, which together contribute to substantial reductions in health-related quality of life. Studies have highlighted that unmet healthcare needs and comorbid depression or functional decline strongly predict lower quality of life and reduced access to appropriate care [24, 25]. Given the chronic and progressive nature of HD, patients may experience a lengthy palliative phase [26], often accompanied by complex care needs [27]. Moreover, evidence from longitudinal registry data suggests that clinical burden and the number of comorbid diagnoses tend to increase with disease duration, especially in patients with more than 10 years of disease progression [28], which further amplifies demands on healthcare and caregiving systems as well as affected families [29].

In Beta-Thalassemia, patients experience a range of comorbidities beyond the anemia itself that significantly shape care needs and resource use. For example, a recent German claims analysis showed that among patients with transfusion-dependent Beta-Thalassemia, endocrine complications, cardiopulmonary issues, and malignancies were common, resulting in very high outpatient utilization and frequent transfusion/routine visits [30]. Another longitudinal real-world study in Europe found that adults with transfusion-dependent Beta-Thalassemia reported substantially lower general health-related quality of life, greater fatigue, pain, and financial burden, and that burden increased with number of comorbidities and poorer hemoglobin control [31]. These comorbidities influence care pathways by necessitating more frequent monitoring, complex multidisciplinary management, and sometimes hospital admissions or complications that require emergent care. They also impair quality of life through physical limitations, and psychosocial impacts, which in turn may reduce adherence and increased need for supportive services.

However, such comorbidities are often underrepresented in disease registries. Systematically capturing this information in administrative data can improve the understanding of multimorbidity patterns and inform the development of more comprehensive, patient-centered clinical guidelines that address the complex needs of individuals living with rare diseases.

Rare diseases pose a significant financial burden on patients and society. In this study, average healthcare costs differed across diseases but increased for all three over time. Joinpoint regression analyses showed that these increases followed consistent linear trends without significant changes in slope, indicating steady cost growth throughout the observation period. The increase was most pronounced for SMA type 1, largely driven by rising medication costs following the introduction of three novel therapeutic interventions in recent years [32]. These treatments offer significant clinical benefits and improved survival, which may also contribute to increased resource use. While gene therapies, such as those recently approved for SMA type 1, entail high upfront costs, their overall value depends heavily on the durability of long-term treatment effects [33, 34]. In terms of direct costs, gene replacement therapy has already been shown to be less costly after 8.25 years compared to chronic treatment regimes [35]. Early evidence from European cohorts suggests that timely diagnosis and initiation of gene therapy can mitigate disease severity and reduce some downstream healthcare needs, potentially offsetting part of the increased initial expenditure [36]. Nevertheless, ongoing supportive care remains essential, but due to the limited availability of long-term follow-up data, the durability of treatment effects—particularly following gene replacement therapy—cannot yet be fully assessed [19, 22, 37]. It can be expected that further advances in the treatment of other rare diseases, especially in the field of gene therapy [38, 39], will drive up healthcare costs in the short term, but will offer substantial long-term benefits for patients.

This study demonstrates the feasibility of using claims data to study rare diseases and provides important epidemiological and economic insights. With the recent implementation of mandatory inpatient Alpha-ID-SE coding and its linkage to ORPHA codes, diagnostic specificity in German claims data is expected to improve, allowing for more accurate identification of rare diseases in future research. While this is an important step forward, full and consistent identification across care settings remains limited as long as outpatient use is not mandatory. Our analysis included only rare diseases with distinct ICD-10-GM codes, which allows for a relatively specific identification of cases in claims data. However, for many other rare diseases without dedicated ICD codes, Alpha-ID-SE coding would significantly improve case detection—especially in the outpatient setting. Since Alpha-ID-SE codes became mandatory in the inpatient sector in April 2023, some improvement in case ascertainment can be expected for inpatient diagnoses. Nevertheless, analyzing rare diseases without a distinct ICD-10-GM code might still result in disease-specific under-ascertainment, which cannot be reliably quantified without external validation data, such as patient registries. In the European context, studies on rare diseases could greatly benefit from the use of common data models (CDMs), which are designed to harmonize disparate data sources across various healthcare systems. Early results using the InGef database, mapped to the Observational Medical Outcomes Partnership (OMOP) CDM format, have successfully replicated the findings presented here [40] and shows promise for facilitating multi-center studies, enabling the testing of data-driven hypotheses on a larger, more diverse population scale. Broader adoption of such frameworks could enhance the comparability and generalizability of research on rare diseases and support international collaboration.

## Limitations

This study offers valuable insights but is subject to limitations inherent to SHI claims data. First, the analysis was restricted to patients with rare diseases that were both diagnosed and documented within the SHI claims data, potentially excluding undiagnosed or miscoded cases. Such under- or miscoding may lead to an underestimation or overestimation of disease prevalence and related healthcare costs. Second, the ICD-10-GM classification system lacks sufficient granularity to capture disease severity or specific subtypes, thereby limiting the precision of clinical differentiation and stratified analyses [41]. Furthermore, the absence of clinical data such as laboratory parameters precludes assessments of disease severity or progression.

Regarding costs, the use of claims data only allows for the assessment of direct medical costs incurred by the healthcare system. Indirect costs—such as productivity losses, informal caregiving, or broader socioeconomic impacts—are not captured and therefore remain unquantified.

## Conclusion

This study provides important data on the prevalence, comorbidities, and economic burden of HD, BT, and SMA, highlighting the challenges of rare disease management. Targeted SHI data analyses support both epidemiological insight and healthcare optimization in the German system.

## Supplementary Information

Below is the link to the electronic supplementary material.

Supplementary Material 1

## Data Availability

The data used in this study cannot be made publicly available in the manuscript, the additional files, or in a public repository due to German data protection laws (Bundesdatenschutzgesetz) and legal reasons. In Germany, the utilization of health insurance data for scientific research is regulated by the Code of Social Law. Researchers must obtain approval from the health insurance providers as well as their responsible authorities. As this approval is given only for a specific research question for a specific time and for a specific group of researchers, data cannot be made publicly available. To facilitate the replication of results, anonymized data used for this study are stored on a secure drive at the Institute for Applied Health Research Berlin GmbH (InGef). Access to the raw data used in this study can only be provided to external parties under the conditions a cooperation contract and can be accessed upon request, after written approval (info@ingef.de), if required.

## Abbreviations

ATC: Anatomical-Therapeutic-Chemical Classification System
BT: Beta-Thalassemia
CDM: Common Data Model
HD: Huntington’s Disease
ICD: 10-GM-International Classification of Disease and Health Related Problems, German Modification, 10th Revision
InGef: Institute for Applied Health Research Berlin GmbH
OMOP: Observational Medical Outcomes Partnership
SHI: Statutory Health Insurance
SMA type 1: Spinal Muscular Atrophy type 1
