# Supplementary material for: Epidemiology and economic burden of selected rare genetic diseases in Germany – a claims database study
Source: Orphanet J Rare Dis. 2025 Nov 28;20:617. doi: 10.1186/s13023-025-04147-8 (PMC12679720; doi:10.1186/s13023-025-04147-8)
Supplement: Supplementary file 1 — Supplementary Material 1 [file 13023_2025_4147_MOESM1_ESM.docx]

Additional File 1 Summary statistics of healthcare costs by sector for the selected rare diseases for the years 2017 to 2023.

| Cost sector | N, summary statistic* | 2017 | 2018 | 2019 | 2020 | 2021 | 2022 | 2023 |
| --- | --- | --- | --- | --- | --- | --- | --- | --- |
| Huntington’s Disease | | | | | | | | |
| Total costs | N | 533 | 565 | 582.00 | 583 | 543 | 583 | 564 |
|  | Sum | 4464207.86 | 4675504.61 | 5458758.68 | 4761120.53 | 5160486.23 | 5544468.12 | 5373729.03 |
|  | Mean | 8375.62 | 8275.23 | 9379.31 | 8166.59 | 9503.66 | 9510.24 | 9527.89 |
|  | SD | 9567.05 | 10899.53 | 13332.66 | 12921.70 | 22821.97 | 13960.17 | 13936.14 |
|  | Min | 177.37 | 72.12 | 108.86 | 107.99 | 86.34 | 166.45 | 101.10 |
|  | Q1 | 1953.39 | 1781.60 | 1868.64 | 1650.02 | 1916.12 | 1952.16 | 1850.79 |
|  | Median | 4982.33 | 4681.60 | 5089.55 | 4303.47 | 4910.31 | 5141.10 | 5258.06 |
|  | Q3 | 11195.82 | 10536.47 | 11942.38 | 9959.72 | 10975.46 | 10965.58 | 11213.13 |
|  | Max | 70861.61 | 106133.76 | 143478.89 | 174197.35 | 437579.36 | 159887.99 | 164874.48 |
| Outpatient costs | Sum | 567357.74 | 561482.08 | 606689.85 | 611505.37 | 593962.41 | 634964.99 | 639213.00 |
|  | Mean | 1064.46 | 993.77 | 1042.42 | 1048.89 | 1093.85 | 1089.13 | 1133.36 |
|  | SD | 1621.65 | 762.99 | 1066.28 | 1115.76 | 1241.78 | 1040.53 | 1140.44 |
|  | Min | 0.00 | 0.00 | 0.00 | 0.00 | 0.00 | 0.00 | 27.11 |
|  | Q1 | 456.78 | 457.29 | 456.00 | 441.22 | 435.75 | 439.84 | 467.85 |
|  | Median | 767.57 | 788.91 | 797.30 | 764.96 | 796.00 | 778.33 | 834.81 |
|  | Q3 | 1264.90 | 1326.52 | 1326.33 | 1326.05 | 1414.93 | 1375.40 | 1406.06 |
|  | Max | 31942.26 | 5225.48 | 17540.14 | 13894.86 | 18769.08 | 7641.25 | 14327.57 |
| Inpatient costs | Sum | 1808996.33 | 1888737.71 | 2370149.17 | 1906471.41 | 2363129.97 | 2416169.16 | 2176029.37 |
|  | Mean | 3393.99 | 3342.90 | 4072.42 | 3270.11 | 4351.99 | 4144.37 | 3858.21 |
|  | SD | 7325.85 | 8077.69 | 11031.11 | 10453.73 | 20677.19 | 10993.92 | 10779.89 |
|  | Min | 0.00 | 0.00 | 0.00 | 0.00 | 0.00 | 0.00 | 0.00 |
|  | Q1 | 0.00 | 0.00 | 0.00 | 0.00 | 0.00 | 0.00 | 0.00 |
|  | Median | 135.00 | 130.09 | 173.88 | 135.00 | 165.00 | 201.01 | 204.03 |
|  | Q3 | 2926.15 | 2890.73 | 2991.48 | 2343.63 | 3106.98 | 2881.15 | 3174.54 |
|  | Max | 61158.41 | 89350.39 | 123727.21 | 164051.08 | 422116.53 | 105704.52 | 126372.76 |
| Medication costs | Sum | 863283.04 | 931282.73 | 924299.11 | 847026.86 | 789660.89 | 990415.43 | 936640.51 |
|  | Mean | 1619.67 | 1648.29 | 1588.14 | 1452.88 | 1454.26 | 1698.83 | 1660.71 |
|  | SD | 2146.53 | 2983.99 | 3162.86 | 2796.36 | 2722.74 | 3685.59 | 3330.61 |
|  | Min | 0.00 | 0.00 | 0.00 | 0.00 | 0.00 | 0.00 | 0.00 |
|  | Q1 | 234.19 | 210.72 | 202.78 | 192.27 | 172.14 | 185.93 | 203.91 |
|  | Median | 780.24 | 741.08 | 728.69 | 668.85 | 657.34 | 668.29 | 700.06 |
|  | Q3 | 2146.77 | 2040.49 | 1851.04 | 1850.09 | 1623.16 | 1864.42 | 1756.33 |
|  | Max | 15245.06 | 47179.39 | 48278.21 | 47895.17 | 35157.29 | 52255.14 | 36810.89 |
| Aids and remedies costs | Sum | 1224570.75 | 1294002.09 | 1557620.55 | 1396116.89 | 1413732.96 | 1502918.54 | 1621846.15 |
|  | Mean | 2297.51 | 2290.27 | 2676.32 | 2394.71 | 2603.56 | 2577.90 | 2875.61 |
|  | SD | 3529.51 | 3783.14 | 4328.49 | 3929.12 | 4842.37 | 4204.56 | 4645.16 |
|  | Min | 0.00 | 0.00 | 0.00 | 0.00 | 0.00 | 0.00 | 0.00 |
|  | Q1 | 0.00 | 0.00 | 0.00 | 0.00 | 0.00 | 0.00 | 0.00 |
|  | Median | 728.23 | 691.50 | 752.99 | 719.68 | 653.49 | 780.86 | 840.85 |
|  | Q3 | 3242.87 | 3164.60 | 3640.99 | 3250.90 | 3190.68 | 3538.58 | 4000.99 |
|  | Max | 21119.78 | 39002.79 | 26615.59 | 35461.22 | 43535.00 | 44551.26 | 38382.19 |
| Beta-Thalassamia | | | | | | | | |
| Total costs | N | 901 | 894 | 923 | 904 | 965 | 1046 | 1072 |
|  | Sum | 4149219.86 | 4263435.72 | 4416873.95 | 4711946.45 | 5329929.69 | 5998795.33 | 7135526.05 |
|  | Mean | 4605.13 | 4768.94 | 4785.35 | 5212.33 | 5523.24 | 5734.99 | 6656.27 |
|  | SD | 11320.79 | 12017.06 | 12151.49 | 14488.78 | 13743.38 | 19269.10 | 18313.49 |
|  | Min | 58.96 | 60.72 | 43.30 | 55.75 | 64.10 | 55.13 | 35.73 |
|  | Q1 | 537.89 | 488.75 | 503.66 | 514.16 | 575.18 | 566.26 | 664.72 |
|  | Median | 1205.89 | 1172.82 | 1224.35 | 1308.92 | 1295.52 | 1364.48 | 1611.09 |
|  | Q3 | 3567.74 | 3630.83 | 3867.25 | 4185.58 | 4447.93 | 4037.34 | 5071.32 |
|  | Max | 146960.76 | 170590.42 | 200305.87 | 271622.49 | 166871.79 | 337705.02 | 207468.16 |
| Outpatient costs | Sum | 843264.96 | 821802.84 | 853347.14 | 934054.14 | 1165615.30 | 1213606.52 | 1382700.78 |
|  | Mean | 935.92 | 919.24 | 924.54 | 1033.25 | 1207.89 | 1160.24 | 1289.83 |
|  | SD | 1146.24 | 1023.55 | 944.55 | 1479.54 | 2157.37 | 1824.79 | 2243.55 |
|  | Min | 0.00 | 28.14 | 43.30 | 0.00 | 9.79 | 37.80 | 0.00 |
|  | Q1 | 341.43 | 324.18 | 348.17 | 351.05 | 383.12 | 393.22 | 432.21 |
|  | Median | 608.82 | 611.75 | 614.80 | 639.03 | 708.15 | 676.30 | 753.83 |
|  | Q3 | 1140.33 | 1136.86 | 1144.37 | 1218.15 | 1293.41 | 1254.45 | 1445.97 |
|  | Max | 16086.77 | 12302.36 | 7570.92 | 24779.96 | 33722.23 | 32498.60 | 33696.53 |
| Inpatient costs | Sum | 1714911.00 | 1808377.91 | 1844228.20 | 1684743.79 | 1682227.81 | 1832127.57 | 2887515.55 |
|  | Mean | 1903.34 | 2022.79 | 1998.08 | 1863.65 | 1743.24 | 1751.56 | 2693.58 |
|  | SD | 8317.57 | 8549.90 | 9481.18 | 9661.55 | 6938.35 | 6586.83 | 11877.25 |
|  | Min | 0.00 | 0.00 | 0.00 | 0.00 | 0.00 | 0.00 | 0.00 |
|  | Q1 | 0.00 | 0.00 | 0.00 | 0.00 | 0.00 | 0.00 | 0.00 |
|  | Median | 0.00 | 0.00 | 0.00 | 0.00 | 0.00 | 0.00 | 0.00 |
|  | Q3 | 624.15 | 403.26 | 712.55 | 566.90 | 312.15 | 390.73 | 517.58 |
|  | Max | 146960.76 | 148300.59 | 184238.98 | 247045.69 | 123891.41 | 89048.41 | 182590.27 |
| Medication costs | Sum | 1288178.44 | 1303509.38 | 1341085.24 | 1732989.33 | 2062159.92 | 2484623.63 | 2291936.73 |
|  | Mean | 1429.72 | 1458.06 | 1452.96 | 1917.02 | 2136.95 | 2375.36 | 2138.00 |
|  | SD | 5601.61 | 5815.02 | 6004.17 | 8511.47 | 10134.11 | 15994.25 | 10650.85 |
|  | Min | 0.00 | 0.00 | 0.00 | 0.00 | 0.00 | 0.00 | 0.00 |
|  | Q1 | 32.92 | 27.98 | 28.52 | 27.87 | 29.02 | 28.40 | 36.68 |
|  | Median | 96.08 | 91.22 | 89.35 | 96.69 | 102.13 | 111.56 | 123.59 |
|  | Q3 | 406.84 | 407.74 | 409.00 | 473.91 | 489.59 | 482.37 | 550.34 |
|  | Max | 82334.41 | 83135.75 | 80813.75 | 145881.69 | 164655.02 | 325422.62 | 202557.22 |
| Aids and remedies costs | Sum | 302865.46 | 329745.59 | 378213.37 | 360159.19 | 419926.66 | 468437.61 | 573372.99 |
|  | Mean | 336.14 | 368.84 | 409.77 | 398.41 | 435.16 | 447.84 | 534.86 |
|  | SD | 1264.68 | 1639.48 | 1587.12 | 1671.71 | 1508.40 | 1775.91 | 2091.01 |
|  | Min | 0.00 | 0.00 | 0.00 | 0.00 | 0.00 | 0.00 | 0.00 |
|  | Q1 | 0.00 | 0.00 | 0.00 | 0.00 | 0.00 | 0.00 | 0.00 |
|  | Median | 0.00 | 0.00 | 0.00 | 0.00 | 0.00 | 0.00 | 0.00 |
|  | Q3 | 191.86 | 180.65 | 216.99 | 180.68 | 219.30 | 234.82 | 275.49 |
|  | Max | 25222.17 | 26960.46 | 31841.86 | 26807.90 | 18886.70 | 30731.08 | 37374.10 |
| Spinal Muscular Atrophy type 1 | | | | | | | | |
| Total costs | N | 90 | 93 | 93 | 97 | 101 | 111 | 104 |
|  | Sum | 4165802.84 | 9431677.58 | 9009414.34 | 18101898.87 | 18343189.20 | 24948301.26 | 15036842.52 |
|  | Mean | 46286.70 | 101415.89 | 96875.42 | 186617.51 | 181615.73 | 224759.47 | 144585.02 |
|  | SD | 84342.37 | 172985.34 | 155365.62 | 460878.43 | 408542.45 | 468087.50 | 256814.73 |
|  | Min | 245.62 | 215.86 | 121.80 | 108.59 | 411.97 | 173.88 | 219.21 |
|  | Q1 | 3080.94 | 3162.91 | 3898.05 | 4275.96 | 4827.78 | 4298.81 | 4786.37 |
|  | Median | 10121.01 | 16188.13 | 20230.14 | 25014.05 | 21795.00 | 35652.97 | 39476.77 |
|  | Q3 | 46597.51 | 97331.44 | 94970.29 | 294545.79 | 271497.86 | 281188.63 | 259463.06 |
|  | Max | 432360.94 | 732143.25 | 708303.00 | 2677655.90 | 2350468.95 | 2422812.34 | 1682863.22 |
| Outpatient costs | Sum | 75739.74 | 93521.83 | 85193.75 | 96799.87 | 100388.43 | 110838.58 | 101665.86 |
|  | Mean | 841.55 | 1005.61 | 916.06 | 997.94 | 993.94 | 998.55 | 977.56 |
|  | SD | 853.25 | 1099.93 | 926.92 | 1192.58 | 1206.85 | 1139.20 | 1025.77 |
|  | Min | 86.89 | 61.79 | 121.80 | 0.00 | 0.00 | 140.78 | 66.94 |
|  | Q1 | 377.55 | 406.02 | 440.34 | 396.24 | 362.21 | 399.23 | 468.44 |
|  | Median | 655.55 | 713.38 | 637.68 | 639.87 | 594.74 | 641.78 | 693.32 |
|  | Q3 | 938.47 | 1080.62 | 1128.00 | 1055.45 | 1114.57 | 1083.35 | 1093.52 |
|  | Max | 6392.35 | 7277.65 | 7099.21 | 7268.30 | 8687.47 | 5876.58 | 6583.75 |
| Inpatient costs | Sum | 2159242.57 | 6482644.08 | 6673903.33 | 15442243.47 | 13858934.48 | 18243116.55 | 8059153.69 |
|  | Mean | 23991.58 | 69705.85 | 71762.40 | 159198.39 | 137217.17 | 164352.40 | 77491.86 |
|  | SD | 69374.51 | 141754.67 | 136162.14 | 458898.59 | 406655.16 | 467164.85 | 245642.92 |
|  | Min | 0.00 | 0.00 | 0.00 | 0.00 | 0.00 | 0.00 | 0.00 |
|  | Q1 | 0.00 | 0.00 | 0.00 | 0.00 | 0.00 | 0.00 | 206.11 |
|  | Median | 2339.57 | 1990.73 | 2199.02 | 3115.47 | 3030.10 | 2484.43 | 2878.57 |
|  | Q3 | 8406.52 | 21107.47 | 19296.66 | 248995.01 | 43262.37 | 52337.45 | 23054.50 |
|  | Max | 418986.38 | 648400.04 | 603562.75 | 2662958.52 | 2334092.32 | 2358080.98 | 1681079.42 |
| Medication costs | Sum | 543777.30 | 831212.60 | 241765.20 | 432653.73 | 2538773.02 | 4908506.59 | 4923752.92 |
|  | Mean | 6041.97 | 8937.77 | 2599.63 | 4460.35 | 25136.37 | 44220.78 | 47343.78 |
|  | SD | 30306.25 | 60973.38 | 9320.43 | 18936.23 | 67744.60 | 97199.37 | 100025.92 |
|  | Min | 0.00 | 0.00 | 0.00 | 0.00 | 0.00 | 0.00 | 0.00 |
|  | Q1 | 87.94 | 57.58 | 90.19 | 65.33 | 38.18 | 94.84 | 93.17 |
|  | Median | 321.01 | 467.67 | 506.76 | 422.88 | 348.29 | 847.00 | 1247.29 |
|  | Q3 | 1970.83 | 2402.13 | 3028.79 | 2430.39 | 3720.02 | 6662.91 | 8126.30 |
|  | Max | 205589.93 | 581170.39 | 88709.05 | 167424.78 | 339583.31 | 354130.48 | 364726.72 |
| Aids and remedies costs | Sum | 1387043.23 | 2024299.07 | 2008552.06 | 2130201.80 | 1845093.27 | 1685839.54 | 1952270.05 |
|  | Mean | 15411.59 | 21766.66 | 21597.33 | 21960.84 | 18268.25 | 15187.74 | 18771.83 |
|  | SD | 25630.55 | 46877.76 | 35607.91 | 36702.82 | 33530.35 | 22364.79 | 36435.09 |
|  | Min | 0.00 | 0.00 | 0.00 | 0.00 | 0.00 | 0.00 | 0.00 |
|  | Q1 | 624.49 | 513.96 | 630.84 | 346.73 | 338.48 | 318.48 | 779.85 |
|  | Median | 3429.14 | 3168.38 | 5116.78 | 5563.35 | 4127.30 | 4349.54 | 6667.80 |
|  | Q3 | 20094.89 | 23550.94 | 33007.28 | 30504.41 | 19740.49 | 23308.72 | 20962.04 |
|  | Max | 155499.79 | 347928.19 | 248146.83 | 259400.79 | 219350.68 | 132626.35 | 269614.10 |
